# The Optimal Hemostasis Duration After Tooth Extraction: A Randomized Controlled Trial

**DOI:** 10.7759/cureus.33331

**Published:** 2023-01-03

**Authors:** Navaneeth Yerragudi, Jitendra G Chawla, Vinoth K Kalidoss, Swapnika Polineni, Cheranjeevi Jayam, Chaitanya Kumar

**Affiliations:** 1 Department of Dentistry, All India Institute of Medical Sciences, Mangalagiri, Mangalagiri, IND; 2 Department of Community and Family Medicine, All India Institute of Medical Sciences, Mangalagiri, Mangalagiri, IND; 3 Department of Transfusion Medicine and Hemotherapy, All India Institute of Medical Sciences, Mangalagiri, Mangalagiri, IND

**Keywords:** tooth extraction, surgical hemostasis, hemostasis, blood clotting, bleeding

## Abstract

Background

There is a lack of evidence-based practice regarding the duration of pressure pack placement following tooth extraction. This study aimed to compare the incidence of post-extraction bleeding following 60 minutes versus 10 minutes of pressure pack placement.

Methodology

A randomized controlled trial was conducted at a tertiary care hospital and included patients requiring intra-alveolar tooth extractions. Patients were randomly allocated into the experimental group or control group by a permuted block randomization method. A blinded observer noted the incidence of post-extraction bleeding. Categorical variables were summarized as frequency and percentage. The chi-square test was used for intergroup statistical analysis. P-values <0.05 were considered statistically significant.

Results

There were 528 participants, 264 of whom were allocated to each group. The incidence of post-extraction bleeding was 8% and 6.8% in the experimental and control groups, respectively. On bivariate analysis, there was no statistically significant difference between the two groups (p = 0.618; relative risk with 95% confidence interval = 1.0).

Conclusions

In the majority of cases, hemostasis was achieved in 10 minutes. Therefore, removing the pressure pack after 10 minutes may be advised to ensure hemostasis and, ultimately, save chairside time.

## Introduction

Tooth extraction is one of the most commonly performed outpatient dental procedures [1]. Bleeding following tooth extraction occurs due to the severing of the periodontal blood vessels, and often such bleeding is controlled by placing a moist cotton gauze pressure pack over the extraction socket [2]. There is a paucity of evidence-based literature describing the optimal duration for which the pressure pack needs to be placed after tooth extraction. In practice, the duration for which the pressure pack is placed over the extraction socket ranges from five minutes to 60 minutes, with no consensus among practitioners [3-6].

Hemostasis from tooth extraction sockets begins with constriction of blood vessels and the formation of platelet plugs that occlude the broken end of vessels. Direct application of pressure is known to aid this process; however, 20 to 60 minutes after tooth extraction, the clot retracts, causing the edges of the broken blood vessel to be pulled together, thus contributing to an ultimate state of hemostasis. Based on the normal range for clot retraction time [7], it has been in practice to ask the patient to bite on the pressure pack for about 60 minutes [8-15]. As the severed blood vessels lining the tooth extraction sockets are not too large, within 10 minutes, which is about the normal clotting time [7], the entire opening of the broken end of the blood vessels is filled with clots. Therefore, some investigators observed that the pressure pack may be removed within five to 10 minutes after tooth extraction [3,16-19]. This difference in practices prompted this study to be conducted to evaluate the optimal time required to achieve hemostasis following tooth extraction. The investigators hypothesize that hemostasis could be achieved with 10 minutes of pressure on the socket following tooth extraction. The specific aim of this study was to compare the incidence of post-extraction bleeding (PEB) following 10 minutes versus 60 minutes of pressure application.

## Materials and methods

This study was approved by the institutional ethics committee vide AIIMS/MG//IEC/2020-21/35 dated September 01, 2020, and registered in the National Clinical Trials Registry. Written informed consent was obtained from all participants. The study adhered to the tenets of the Declaration of Helsinki and followed the recommendations of the CONSORT statement for reporting randomized controlled trials.

Study design/sample

To address the research purpose, the investigators designed and implemented a parallel-arm randomized controlled clinical trial. The study population was composed of all patients presenting to the Department of Dentistry of a tertiary care hospital for evaluation and management of dental caries, periodontal disease, malpositioned teeth, impacted teeth, and requiring tooth extraction between December 2020 and December 2021. To be included in the study, patients needed to be aged above 18 years and required intra-alveolar extraction. Patients were excluded if they required a trans-alveolar extraction, were on any anti-platelet and/or anti-coagulant medication, had a preoperative blood pressure of >160/90 mmHg, had jaundice at presentation, and had known bleeding or clotting disorders.

Based on a cross-sectional study by Kumar et al. [3], where the incidence of bleeding following pressure pack removal after 10 minutes was 3%, while the incidence of bleeding after 60 minutes of pressure pack application was 0.003% with a confidence interval of 95% and a power of 80%, the sample size was calculated as 264 in each group using OpenEpi® 3.01. A consecutive sampling method was used, and patients were randomly assigned using permuted block randomization using computer-generated blocks of 16. An intention-to-treat analysis was done.

Study variables

The primary predictor variable was the duration for which the pressure pack was placed after extraction. The primary outcome variable was the incidence of PEB, defined as evidence of bleeding beyond the pressure pack [20]. The co-variables included incidence of reactionary bleeding (delayed hemorrhage occurring within 24 hours), secondary hemorrhage (hemorrhage occurring seven to 10 days after tooth extraction) [9], and alveolar osteitis characterized by postoperative pain inside and around the extraction site with an increase in severity between the first and third day after extraction accompanied by a partial or total disintegrated blood clot within the extraction socket with or without halitosis [21].

Data collection methods

After extraction, the surgeon opened an opaque envelope that determined the allocation to either the experimental or control group. Based on the allocation, a blinded observer was asked to assess PEB after the required duration determined by the allotment (10 minutes in the experimental group versus 60 minutes in the control group). The blinded observer first asked the patient to swallow the pooled saliva and then keep the mouth open while the observations were made.

If PEB was evident, a fresh pack was placed and the socket was inspected again after 10 minutes. If PEB was noted even after the second pack, a fresh gauze pack soaked in 1 g of tranexamic acid (hemostatic pack) was placed for 30 minutes. If bleeding persisted beyond this point, it was planned to suture the socket margins, place a hemostatic pack, and investigate the patient. Patients were discharged with routine verbal and written post-extraction instructions. The observer noted the findings and attached them to the patient’s records.

All patients were followed up for incidents of reactionary hemorrhage and secondary hemorrhage by questioning the need for at-home pressure pack placement via a telephone interview 24 hours, four days, and seven days after the procedure. Standard alveolar osteitis assessment criteria were used which included radiating pain and clinical examination [4].

Data analysis

Data were analyzed using Epi-info® and R® software. Categorical variables were summarized as frequency and percentage. The association between categorical variables was assessed using the chi-square test. Binary logistic regression was used to estimate the adjusted odds ratio which was reported with a 95% confidence interval. A p-value less than 0.05 was considered statistically significant.

## Results

**Figure 1:**
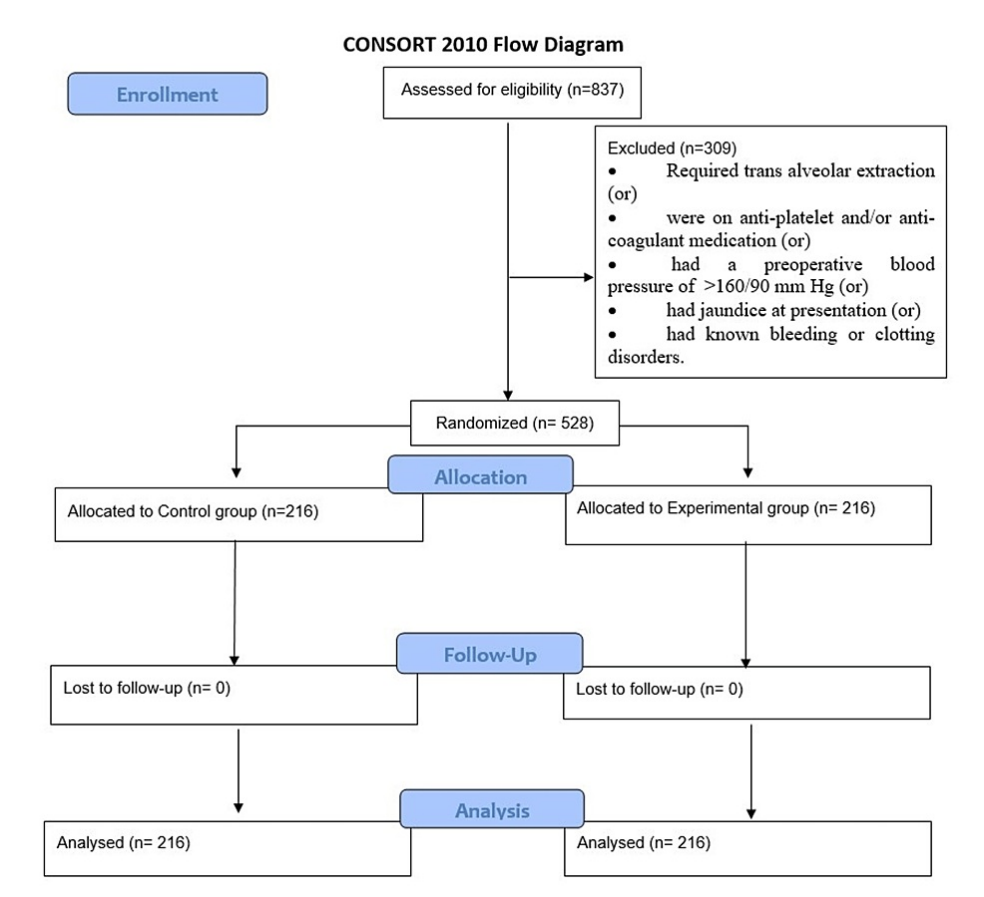
CONSORT flow diagram.

A total of 837 patients were screened for eligibility, and 528 patients were enrolled as study subjects with no loss of subjects to follow up (Figure 1).

The experimental group and control group comprised 264 participants in each group, with no statistically significant difference in age (p = 0.30), gender (p = 0.93), the presence of pus discharge from periodontal tissues (p = 0.12), the arch in which the tooth was located (p = 0.43), the type of tooth (p = 0.16), and mobility of tooth (p = 0.54). Further, the two groups were also similar in the instruments used for extraction among the patients (p = 1.00) (Table 1).

**Table 1:** Patient characteristics.

Item	Subgroup	Experimental group, n (%)	Control group, n (%)	P-value
Age group	18–40 years	85 (45.5)	102 (54.5)	0.3
	41–60 years	126 (52.3)	115 (47.7)	
	>60 years	53 (53.0)	47 (47.0)	
Gender	Male	108 (49.8)	109 (50.2)	0.93
	Female	156 (50.2)	155 (49.8)	
Pus discharge from periodontal tissues	Present	10 (35.7)	18 (64.3)	0.12
	Absent	254 (50.8)	246 (49.2)	
Arch in which the tooth is located	Mandible	120 (51.9)	111 (48.1)	0.43
	Maxilla	144 (48.5)	153 (51.5)	
Type of tooth	Molar	200 (52.6)	180 (47.4)	0.164
	Premolar	40 (43.5)	52 (56.5)	
	Incisor	12 (36.4)	21 (63.6)	
	Canine	12 (52.2)	11 (47.8)	
Grace and Smale’s grading of mobility of tooth	0	191 (51.6)	179 (48.4)	0.543
	1	13 (39.4)	20 (60.6)	
	2	16 (50.0)	16 (50.0)	
	3	44 (47.3)	49 (52.7)	
Instruments used for tooth extraction	Elevator	47 (51.1)	45 (48.9)	1
	Forceps	133 (50.2)	132 (49.8)	
	Elevator + forceps	84 (49.1)	87 (50.9)	

Univariate and multivariate analysis between the patient characteristics and incidence of PEB showed that the incidence of PEB was higher with the presence of infection (adjusted OR = 7.6 compared to the absence of pus discharge) and the use of both elevators and forceps for extraction (adjusted OR = 3.1 compared to the use of forceps alone) (Table 2).

**Table 2:** Comparison of patient characteristics and the incidence of PEB. PEB = post-extraction bleeding; OR = odds ratio; CI = confidence interval; Ref = reference group

Item	Subgroup	PEB absent, n (%)	PEB present, n (%)	Unadjusted OR (95% CI)	Adjusted OR (95% CI)
Age group	18–40 years	178 (95.2)	9 (4.8)	Ref	Ref
	41–60 years	218 (90.5)	23 (9.5)	2.1 (0.9-4.6)	1.3 (0.4-4.7)
	>60 years	93 (93.0)	7 (7.0)	1.5 (0.5-4.1)	0.5 (0.2-1.5)
Gender	Male	195 (89.9)	22 (10.1)	1.9 (1.01-3.7)	2.2 (1.0-4.9)
	Female	294 (94.5)	17 (5.5)	Ref	Ref
Pus discharge from periodontal tissues	Present	20 (71.4)	8 (28.6)	6.1 (2.5-14.8)	7.6 (2.5-23.2)
	Absent	469 (93.8)	31 (6.2)	Ref	Ref
Arch in which the tooth is located	Mandible	215 (93.1)	16 (6.9)	Ref	Ref
	Maxilla	274 (92.3)	23 (7.7)	1.1 (0.5-2.1)	1.5 (0.7-3.3)
Type of tooth	Molar	350 (92.1)	30 (7.9)	2.7 (0.4-20.7)	0.2 (0.0-2.4)
	Premolar	88 (95.7)	4 (4.3)	1.4 (0.1-13.5)	0.6 (0.7-5.8)
	Canine	19 (82.6)	4 (17.4)	6.7 (0.7-64.7)	1.4 (0.1-15.4)
	Incisor	32 (97.0)	1 (3.0)	Ref	Ref
Grace and Smale’s grading of mobility of tooth	0	346 (93.5)	24 (6.5)	Ref	Ref
	1	27 (81.8)	6 (18.2)	3.2 (1.2-8.5)	0.4 (0.1-1.3)
	2	30 (93.8)	2 (6.3)	0.9 (0.2-4.2)	1.1 (0.2-7.1)
	3	86 (92.5)	7 (7.5)	1.2 (0.5-2.8)	0.5 (0.1-2.4)
Instruments used for tooth extraction	Forceps	253 (95.5)	12 (4.5)	Ref	Ref
	Elevator	85 (92.4)	7 (7.6)	1.7 (0.7-4.5)	1.3 (0.5-3.7)
	Elevator + forceps	151 (88.3)	20 (11.7)	2.8 (1.3-5.9)	3.1 (1.2-8.7)

PEB was noted in 8% of patients in the experimental group (n = 21) and 6.8% of patients in the control group (n = 18). Bivariate analysis showed that there was no statistically significant difference between the two groups. (p = 0.618, relative risk with 95% confidence interval = 1.0). Following the use of an additional pack, PEB was noted in 0.4% (n = 1) of patients in the experimental group versus 1.5% (n = 4) of patients in the control group, with no statistically significant difference between the groups (p = 0.287) (Table 3).

**Table 3:** Comparison of PEB between the experimental group and control group. PEB = post-extraction bleeding; RR = relative risk; CI = confidence interval

Item	Experimental group, n (%)	Control group, n (%)	P-value	RR (95% CI)
PEB following pack removal	21 (8.0)	18 (6.8)	0.618	1.0 (0.8-1.4)
PEB following additional pressure pack	1 (0.4)	4 (1.5)	0.287	0.2 (0.02-1.7)

None of the patients required any measures other than the use of a hemostatic pack to achieve hemostasis. There were no incidences of reactionary hemorrhage, secondary hemorrhage, and/or alveolar osteitis among the entire study population.

## Discussion

Based on the understanding of the physiology of hemostasis, this study aimed to evaluate the incidence of PEB using a pressure pack for two different time durations, i.e., 10 minutes versus 60 minutes, and thus estimate the optimal hemostasis duration. The specific aims of the study were to assess the incidence of PEB, the incidence of reactionary hemorrhage, secondary hemorrhage, and alveolar osteitis.

The results of this study show that adequate hemostasis was achieved after 10 minutes of pressure pack placement with no statistically significant difference in the incidence of PEB between the two groups. Further, the incidence of reactionary hemorrhage and secondary hemorrhage was also noted to be similar between the two groups. There was no incidence of alveolar osteitis in any of the patients included in the study.

There is no evidence-based literature describing the duration for which the pressure pack needs to be placed for achieving hemostasis after tooth extraction. Although multiple studies are being performed to devise policies on the management of patients with anti-platelet/anti-coagulant drugs requiring dental extractions, none of them mention a standard duration for which a pressure pack is placed over the socket following tooth extraction [12,15,18]. The study findings are consistent with those of Kumar et al., who conducted a cross-sectional study to quantify the average time required for hemostasis to occur in an extraction socket and found that hemostasis occurred in less than 10 minutes in 96.5% of their cases [3]. There was no incidence of dry sockets in any of the patients included in this study. This could be attributed to the delivery of detailed post-extraction instructions to each patient both verbally and in the form of written instructions before the patient was sent home, similar to the results obtained from the study conducted by Alsaleh et al. [22].

Conventionally, patients get discharged with instructions to remove the pressure pack by themselves after about an hour. Instances of PEB following pressure pack removal are frequent complications, causing patients to return to the clinic [23]. This can be avoided if the pressure pack can be removed and hemostasis can be ensured before the patient’s discharge from the clinic. If the bleeding from a tooth extraction socket would stop in 10 minutes, it may be advisable that all patients who undergo dental extractions have their sockets checked for hemostasis before discharge.

The strength of the present study is the fact that it is the first randomized controlled clinical trial to estimate the optimal hemostasis duration after tooth extraction. Moreover, the follow-up period was designed in such a way that it identified any incidences of PEB, reactionary bleeding, and secondary bleeding in both groups. However, there were shortcomings which included the limitation of inclusion criteria to patients with no hemorrhagic tendencies and a subjective assessment of hemostasis. Future multicentric studies may be planned considering these limitations.

## Conclusions

There was no difference in the incidence of PEB between the test group and the control group. Hence, it is advisable to discontinue the pressure pack after 10 minutes following its placement and ensure hemostasis before the patient is discharged. This will help avoid incidences of return to the clinic or emergency room with PEB.
